# Secondary bladder and colorectal cancer after treatments for prostate cancer: A population based study

**DOI:** 10.1002/cam4.6922

**Published:** 2024-02-13

**Authors:** Patricia Grisel Quintana Bárcena, Armen Garo Aprikian, Alice Dragomir

**Affiliations:** ^1^ Urology, Department of Surgery McGill University Montreal Quebec Canada; ^2^ Research Institute of the McGill University Health Centre Montreal Quebec Canada; ^3^ McGill University Health Centre Montreal Quebec Canada; ^4^ Faculty of pharmacy University of Montreal Montreal Quebec Canada

**Keywords:** androgen deprivation therapy, bladder cancer, brachytherapy, colorectal cancer, external beam radiation therapy, prostate cancer, secondary malignancies

## Abstract

**Background:**

Prostate cancer (PCa) patients receiving radiotherapy may be predisposed to secondary malignancies. This study aimed to determine the association between PCa treatments, including radical prostatectomy (RP), external beam radiation therapy (EBRT), brachytherapy (BT) and androgen deprivation therapy (ADT); and secondary bladder and colorectal cancer.

**Methods:**

A cohort study was constructed using Quebec administrative databases (Med‐Echo and RAMQ). Included men were diagnosed and treated for PCa between 2000 and 2016. Patients with bladder or colorectal cancer prior to PCa were excluded. Follow‐up ended at the earliest of the following: incidence of bladder or colorectal cancer, death, or December 31, 2016. EBRT, BT, EBRT+ADT, RP + ADT or ADT only were compared individually to RP. The incidence of secondary bladder and colorectal cancer were computed. Inverse probability of treatment weighting (IPTW) based on a propensity score was used to control for potential confounding. IPTW‐Cox proportional hazards models were used.

**Results:**

A significant association was found between secondary bladder cancer and EBRT (HR: 1.84, 95%CI: 1.60;2.13), and also EBRT+ADT (HR: 2.08, 95%CI: 1.67;2.56), but not with BT (HR: 1.36, 95%CI: 0.68;2.74). Secondary colorectal cancer was significantly associated to either EBRT (HR: 1.36, 95%CI: 1.21;1.53); or BT (HR: 2.46, 95%CI: 1.71;3.54). The association between ADT alone and both secondary cancers was also significant (HR: 1.98, 95%CI: 1.69;2.31 and HR: 1.69, 95%CI: 1.49;1.92, respectively).

**Conclusions:**

Compared to PCa patients undergoing RP, the secondary bladder cancer was associated with EBRT, ADT, alone or in combination. The secondary colorectal cancer was also associated with receiving either EBRT, BT or ADT.

## Graphical abstract

This study evaluated the relationship between treatments given for prostate cancer including surgery, external beam radiotherapy, brachytherapy and androgen deprivation therapy; and the development of secondary bladder and colorectal cancers in a large Canadian population. Compared to surgery, the secondary bladder cancer risk is higher in patients receiving external beam radiotherapy or hormone therapy. The secondary colorectal cancer risk is also higher in patients receiving external beam radiotherapy, brachytherapy, or hormone therapy.

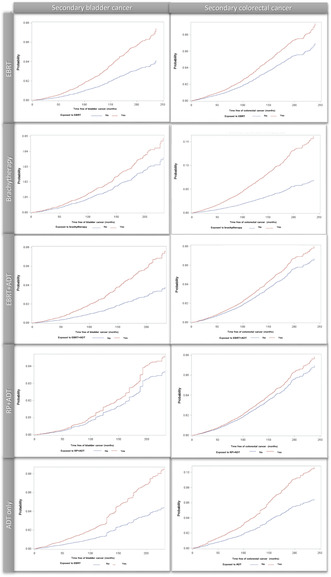

## 1 INTRODUCTION

Prostate cancer (PCa) is the most common type of cancer in Canadian men, followed by colorectal, lung, and bladder; and the third deadliest cancer (9.6% of cancer‐related deaths).^1^ According to the Canadian Cancer Society, 1 in 8 men will receive a PCa diagnosis during their lifetime and 1 in 29 will die from the disease.^2^

Initial treatments for PCa include radical prostatectomy (RP), radiotherapy (RT), or androgen deprivation therapy (ADT).^3^ Common side effects of RP and RT include urinary and erectile dysfunction. RT is also associated with quality‐of life‐detriments affecting adjacent organs (i.e., bladder and rectum).^4^, ^5^ Common adverse effects include pelvic pain, lower urinary tract symptoms (i.e., dysuria, incontinence, and hematuria), and rectal symptoms (i.e., tenesmus, hematochezia, fistula, and ulceration).^6^ Additionally, an increased prevalence of secondary malignancies in men who received RT has been reported.^7^, ^8^ Either an increased risk^9^ or no association^7^ have been described depending on the affected organ. Among them, the bladder is the most frequent site for secondary cancer in the radiation field followed by rectal cancer.^10^ PCa patients receiving RT have a nearly 50% increased risk of bladder cancer compared with the non‐radiation group.^11^ Pooled hazard ratios (HR) have shown an increased risk for colorectal cancer in those treated with RT compared to other treatments (three studies; adjusted HR: 1.79, 95% confidence interval [CI]: 1.34;2.38).^12^ While these results indicate a consistent trend, several meta‐analyses exclude more recent data. Moreover, individual studies might have a risk of bias since they have not accounted for baseline differences between patients undergoing RT and RP, including age and comorbidities.

Regarding ADT, it is deemed a first‐line treatment option for men with metastatic PCa alone or more recently, in combination with chemotherapy. A prolonged suppression of androgens has been associated with a condition called the “androgen deprivation syndrome”, characterized by dyslipidemia, insulin resistance, and increased fat mass, which have all been identified as risk factors of colorectal cancer.^13^, ^14^ Furthermore, androgens have shown a protective effect on colorectal carcinogenesis.^15^ An increased risk of colorectal cancer has been reported with the use of gonadotropin‐releasing hormone (GnRH) agonists (HR: 1.31, 95%CI: 1.12;1.53),^13^ and bilateral orchiectomy (HR: 2.50, 95%CI: 1.13;5.52).^14^ While the evidence is limited, the overall risk associated with ADT remains unknown.^14^ Given the recent evidence supporting an early use of ADT,^16^ it is important to adequately assess the association between ADT and colorectal cancer.

In this retrospective study using a Canadian provincial database, the objective was to determine the association between exposure to different treatments for PCa, including RP, external beam radiation therapy (EBRT), brachytherapy (BT) and ADT, (alone or combined), and the development of subsequent secondary bladder and colorectal malignancies.

## 2 PATIENTS AND METHODS

We conducted an observational retrospective cohort study using data from the Régie de l'Assurance Maladie du Québec (RAMQ) and the Maintenance et exploitation des données pour l'étude de la clientèle hospitalière (MED‐ECHO) databases, which are used to administer the public health care insurance programs in Quebec. The MED‐ECHO database contains information about acute‐care hospitalizations (date of admission, length of stay, and diagnoses). The RAMQ is subdivided in four database types: (1) The beneficiary database, containing age, sex, social assistance status, and date of death; (2) the medical services dataset, containing claims for inpatient and ambulatory services; date, nature and location of the medical services; diagnoses and procedure codes, plus associated costs; (surgical procedure codes conform to the Canadian classification of diagnostic, therapeutic, and surgical procedures); (3) the pharmaceutical database, containing data about medications dispensed in community pharmacies, including date, drug name, dose, quantity, dosage form, duration, and drug costs (both insured and/or paid by patients); and (4) the admissibility database, listing eligibility periods for the RAMQ's public insurance plan. All databases were linked through the individual's health insurance number, serving as unique identifier. No written informed consent was necessary for this study, as it was based on information extracted from user files in both databases without interactions with the research participants in accordance with the provisions of Section 21 of the Act respecting the protection of personal information in the private sector (Lois refondues du Québec [L.R.Q] c. P‐39.1) and Section 125 of the Act respecting access to documents held by public bodies and the protection of personal information (L.R.Q c. A‐2.1). Approval for this study was obtained from the McGill University Health Center Ethics Board and the Comission d'accès à l'information du Québec before data was obtained from the databases.

### 2.1 Study cohort

We identified in the RAMQ databases adult male patients having a PCa diagnosis code, a PCa related procedure code (RP, EBRT, BT, or prostate needle biopsy), or initiating ADT as identified from the RAMQ medical services database, pharmaceutical or MED‐ECHO, from January 2000 to December 2016. The initial treatment date (RP, EBRT, BT, RP + ADT, EBRT+ADT, ADT only) was deemed as the index date. We excluded patients with no prostate biopsy within a 3‐month period before the initial treatment. Time to secondary cancer was calculated from the date receiving any of the aforementioned treatments to the earliest of the following: First date of secondary cancer diagnosis (bladder or colorectal), death, or end of the study period (December 31, 2016).

### 2.2 Outcomes

The outcomes of interest were the occurrence of a primary cancer tumor^17^ of the bladder or colorectal after the index date. These were identified from RAMQ databases by specific diagnostic codes, as follows: bladder cancer: ICD‐10 codes C670–C679, C680, C681, C688 and C689; and colorectal: ICD‐10 codes C182–C189, C19, C210–C212, and C218. Patients with history of one or more of the addressed endpoints before index date were excluded.

### 2.3 Covariates

Among covariates were included: age at index date and comorbidity score 1‐year prior to index date, as these might be associated with the choice of received treatment and be risk factors for the study outcomes.^18^, ^19^, ^20^ The comorbidity score, serving as a proxy of overall health status, was estimated by a patient's Charlson score^21^ in the 1‐year period preceding the index date. In addition, comorbidities were identified from medical and MED‐ECHO databases by specific diagnosis codes and procedures in the 3‐year period prior to the index date, including cancer prior to PCa, cardiovascular events and chronic diseases such as: diabetes, hypertension, and dyslipidemia, which are known factors associated with mortality. Chronic diseases were defined by diagnostic codes, as follow: Diabetes: ICD‐9 code 250 or ICD‐10 code E10–E14; dyslipidemia: ICD‐9 code 272 or ICD‐10 code E78, hypertension: ICD‐9 code 401 and ICD10 code I10.

### 2.4 Statistical analyses

Descriptive statistics were presented as counts and percentages for categorical variables, and as means for continuous variables. Crude incidence rates (IR) were reported as number of events per 100 person‐years (100PYs).

To account for potential baseline confounding due to the distribution differences in baseline characteristics, inverse probability of treatment weighting (IPTW)^22^ was used to create a “pseudo‐population” from the original study population, in which treatment assignment is independent of the measured baseline confounders.^23^ A propensity score was derived through a multivariable logistic regression model estimating the probability of being treated with RP compared to EBRT, BT, RP + ADT, EBRT+ADT or ADT only, given the baseline characteristics (covariates) mentioned above for each patient. An IPTW was derived from the propensity score and then applied to Cox proportional hazards regression models to estimate cause‐specific HR with corresponding 95%CI for secondary bladder and colorectal cancer. Using this approach, the treatment groups were balanced in baseline covariates, and all patients contributed to the final analysis with their respective calculated weights. For each individual, a stabilized weight was obtained by multiplying the inverse probability of treatment by the marginal probability of receiving the actual treatment received. The propensity score was based on demographic and clinical characteristics evaluated at the index date, i.e., age over 75 years (yes vs. no), prior cardiovascular disease events (2 or more vs. less than 2), hypertension (yes vs. no), dyslipidemia (yes vs. no), diabetes (yes vs. no), and Charlson comorbidities score (4 or more vs. less than 4). Finally, absolute standardized mean differences (ASMD) were used to assess balance in baseline characteristics between groups (RP vs. EBRT, BT, RP + ADT, EBRT+ADT, and ADT only) in the unweighted and weighted cohorts. ASMD values greater than 10% are considered evidence of covariate imbalance.^24^

The Cox proportional hazards regression models were used to evaluate the association between the treatment received (EBRT, BT, EBRT+ADT, RP + ADT or ADT only, compared individually to RP) and secondary bladder or colorectal cancers in each weighted cohort. The proportional hazards assumption was investigated graphically based on the scaled Schoenfeld residuals. Crude and IPTW adjusted HR were computed with corresponding 95%CI. Weighted cumulative incidence functions (CIF) were plotted and 12‐month risks were extracted. Since all baseline characteristics were balanced in the weighted cohort, regressions only included the treatment variable.

Analyses were performed using the SAS software (version 9.4: SAS Institute, Cary, NC, USA). All tests were two‐sided, with a significance threshold of 5%.

## 3 RESULTS

The cohort consisted of a total of 63,411 men of whom 15,544, 1229, 4463, 831, 13,506, and 27,838 patients, treated with EBRT, BT, EBRT+ADT, RP + ADT, ADT only and RP, respectively. Among these, 209 and 188 patients presented evidence of bladder and colorectal cancers respectively, prior to the index date and were excluded for the analyses. Mean age (years old) by treatment was: ADT only: 75.9; EBRT+ADT: 71.8; EBRT: 70.3; BT: 67.2; RP + ADT: 66.2; and RP: 63.7. Patients' baseline characteristics in each unweighted and weighted cohort are presented in Table 1.

**TABLE 1:** Cohort baseline characteristics for prostate patients with secondary bladder and colorectal cancer.

Study cohort		Treatment	*N*	Variables were present in the cohort						
				Age >75 years (yes/no)	Prior cardiovascular disease (2 or more vs. less than 2)	Prior cancer (yes/no)	Prior hypertension (yes/no)	Prior dyslipidemia (yes/no)	Prior diabetes (yes/no)	Charlson comorbidities score (4 or more vs. less than 4)
Secondary bladder cancer	Crude/unweighted	EBRT	15,481	27.7	38.9	9.1	62.9	43.2	20.0	49.0
		RP	27,783	1.9	27.2	8.3	49.2	37.9	13.6	28.1
		ASMD before IPTW		0.78	0.117	0.0147	0.137	0.052	0.064	0.209
	IPT‐weighted	EBRT	15,481	11.2	31.0	7.7	54.4	39.6	15.9	35.6
		RP	27,783	11.8	31.9	37.1 8.6	54.5	40.2	15.9	36.2
		ASMD after IPTW		0.015	0.008	0.004	0.004	0.005	0.000	0.006
	Crude/unweighted	BT	1225	14.3	31.7	6.7	61.4	50.7	20.5	48.4
		RP	27,783	1.9	27.2	7.7	49.2	37.9	13.6	28.1
		ASMD before IPTW		0.123	0.045	0.009	0.123	0.127	0.068	0.203
	IPT‐weighted	BT	1225	2.4	26.1	7.8	50.7	36.6	13.3	28.6
		RP	27,783	2.5	27.3	7.6	49.7	38.5	13.9	28.9
		ASMD after IPTW		0.001	0.012	0.001	0.005	0.019	0.006	0.003
	Crude/unweighted	EBRT + ADT	4454	33.3	40.9	7.9	68.3	49.9	22.2	57.5
		RP	27,783	1.9	27.2	7.7	49.2	37.9	13.6	28.1
		ASMD before IPTW		0.313	0.137	0.003	0.191	0.119	0.085	0.294
	IPT‐weighted	EBRT + ADT	4454	6.4	27.7	8.1	51.9	39.9	13.9	32.7
		RP	27,783	6.7	29.4	7.9	52.2	39.8	14.7	32.6
		ASMD after IPTW		0.002	0.017	0.002	0.003	0.001	0.008	0.001
	Crude/unweighted	RP + ADT	827	4.1	32.0	36.4	54.3	39.9	14.2	38.9
		RP	27,783	2.7	27.2	7.7	49.2	37.9	13.6	28.1
		ASMD before IPTW		0.021	0.049	0.287	0.051	0.019	0.005	0.109
	IPT‐weighted	RP + ADT	827	2.0	26.7	8.8	48.37	37.6	12.4	29.7
		RP	27,783	2.1	27.3	8.5	49.3	38.0	13.7	28.4
		ASMD after IPTW		0.006	0.007	0.003	0.010	0.004	0.012	0.129
	Crude/unweighted	ADT only	13,432	61.5	47.6	20.4	71.3	44.5	22.3	63.3
		RP	27,783	1.9	27.2	48.7	49.2	37.9	13.6	28.1
		ASMD before IPTW		0.595	0.205	0.282	0.221	0.065	0.086	0.352
	IPT‐weighted	ADT only	13,432	21.2	32.9	39.9	56.3	40.0	15.9	39.5
		RP	27,783	21.1	33.7	40.5	56.8	40.7	16.1	40.7
		ASMD after IPTW		0.001	0.007	0.005	0.005	0.007	0.002	0.012
Secondary colorectal cancer	Crude/unweighted	EBRT	15,499	27.8	38.9	9.2	62.9	43.2	20.0	49.0
		RP	27,788	2.0	27.2	7.7	49.2	38.0	13.7	28.1
		ASMD before IPTW		0.258	0.117	0.015	0.137	0.051	0.063	0.209
	IPT‐weighted	EBRT	15,499	11.3	31.6	8.3	54.1	39.6	15.9	35.6
		RP	27,788	11.7	31.9	8.7	54.5	40.2	16.0	36.3
		ASMD after IPTW		0.005	0.009	0.004	0.0054	0.00611	0.000	0.006
	Crude/unweighted	BT	1223	14.4	31.8	6.6	61.6	50.8	20.4	48.3
		RP	27,788	2.0	27.2	7.7	49.2	38.0	13.7	28.1
		ASMD before IPTW		0.124	0.046	0.011	0.124	0.128	0.068	0.202

	IPT‐weighted	BT	1223	2.4	26.1	7.8	49.3	36.6	13.3	28.6
		RP	27,788	2.5	27.4	7.6	49.7	38.5	13.9	28.9
		ASMD after IPTW		0.004	0.013	0.002	0.004	0.019	0.006	0.004
	Crude/unweighted	EBRT + ADT	4445	33.3	40.9	7.8	68.2	49.8	22.1	57.4
		RP	27,788	2.0	27.2	7.7	49.2	38.0	13.7	28.1
		ASMD before IPTW		0.313	0.137	0.001	0.190	0.118	0.003	0.293
	IPT‐weighted	EBRT + ADT	4445	6.4	27.7	8.2	51.9	39.9	14.0	32.8
		RP	27,788	6.7	29.4	7.9	52.2	39.8	14.8	32.6
		ASMD after IPTW		0.003	0.017	0.003	0.002	0.019	0.019	0.002
	Crude/unweighted	RP + ADT	831	4.1	32.0	36.7	54.4	39.9	14.2	38.9
		RP	27,788	2.0	27.2	7.7	49.2	38.0	13.7	28.1
		ASMD before IPTW		0.048	0.048	0.290	0.019	0.003	0.006	0.109
	IPT‐weighted	RP + ADT	831	2.7	26.8	8.8	48.4	37.8	12.5	29.7
		RP	27,788	2.1	27.3	8.5	49.3	38.1	13.7	28.4
		ASMD after IPTW		0.0062	0.006	0.003	0.003	0.007	0.0012	0.013
	Crude/unweighted	ADT only	13,437	61.5	47.6	13.5	71.3	44.5	22.3	63.3
		RP	27,788	2.0	27.2	7.7	49.2	38.0	13.7	28.1
		ASMD before IPTW		0.595	0.204	0.059	0.221	0.651	0.086	0.352
	IPT‐weighted	ADT only	13,437	21.6	32.4	10.3	56.3	39.8	15.8	39.9
		RP	27,788	24.3	34.9	13.5	57.8	41.4	16.5	41.9
ASMD after IPTW		0.027	0.025	0.027	0.015	0.016	0.007	0.019

Abbreviations: ASMD, absolute standardized mean differences; BT, brachytherapy; EBRT, external beam radiotherapy; IPTW, inverse probability of treatment weighting; RP, radical prostatectomy.

For the unweighted cohorts, most patients receiving treatments other than RP, were older and had more comorbidities. After applying the IPTW method, the treatment groups were well balanced for all covariates, as showed by the ASMD.

The results of the Cox proportional hazards (Table 2) applied in the weighted cohorts showed that compared to RP, the risk of secondary bladder cancer increases with EBRT and EBRT+ADT (HR: 1.84, 95%CI: 1.60;2.13 and HR:2.08, 95%CI: 1.67;2.56), but not with BT (HR:1.36, 95%CI:0.68;2.74). While for secondary colorectal cancer, an increased risk is observed for patients undergoing either EBRT (HR: 1.0.36, 95%CI: 1.21;1.53) or BT (HR: 2.46, 95%CI: 1.71;3.54).

**TABLE 2:** Risk of secondary malignancies (bladder or colorectal cancer) for patients exposed to different treatments.

		Events	PYs	Incidence rate (IR)/100 PYs	Hazard ratio (HR) 95%CI		12‐month risk (IPTW)
					Crude	IPTW adjusted	
Bladder cancer	RP	344	261,194.67	0.13	Ref.		0.21
	EBRT	368	127,889	0.29	2.27 (1.96;2.64)	1.84 (1.60;2.13)	0.38
	Brachytherapy	11	5647.75	0.19	2.03 (1.11;3.72)	1.36 (0.68;2.74)	0.25
	EBRT + ADT	114	34,676	0.33	2.63 (2.13;3.25)	2.08 (1.67;2.56)	0.39
	RP + ADT	12	7474.08	0.16	1.20 (0.68;2.13)	1.25 (0.68;2.30)	0.20
	ADT only	239	75,720.25	0.32	2.80 (2.37;3.31)	1.98 (1.69;2.31)	0.44
Colorectal cancer	RP	709	260,030.08	0.27	Ref.		0.35
	EBRT	487	127,441.91	0.38	1.44 (1.29;1.62)	1.36 (1.21;1.53)	0.47
	Brachytherapy	27	5576.5	0.48	2.36 (1.60;3.47)	2.46 (1.71;3.54)	0.81
	EBRT + ADT	128	34,656.25	0.37	1.41 (1.17;1.71)	1.20 (0.99;1.46)	0.40
	RP + ADT	30	7432.42	0.40	1.47 (1.02;2.11)	1.13 (0.77;1.68)	0.39
	ADT only	320	75,595.5	0.42	1.78 (1.56;2.03)	1.69 (1.49;1.92)	0.54

Abbreviations: ADT, androgen deprivation therapy; EBRT, external beam radiotherapy; IPTW, inverse probability of treatment weighting; PYs, person‐years; RP, radical prostatectomy.

**FIGURE 1:**
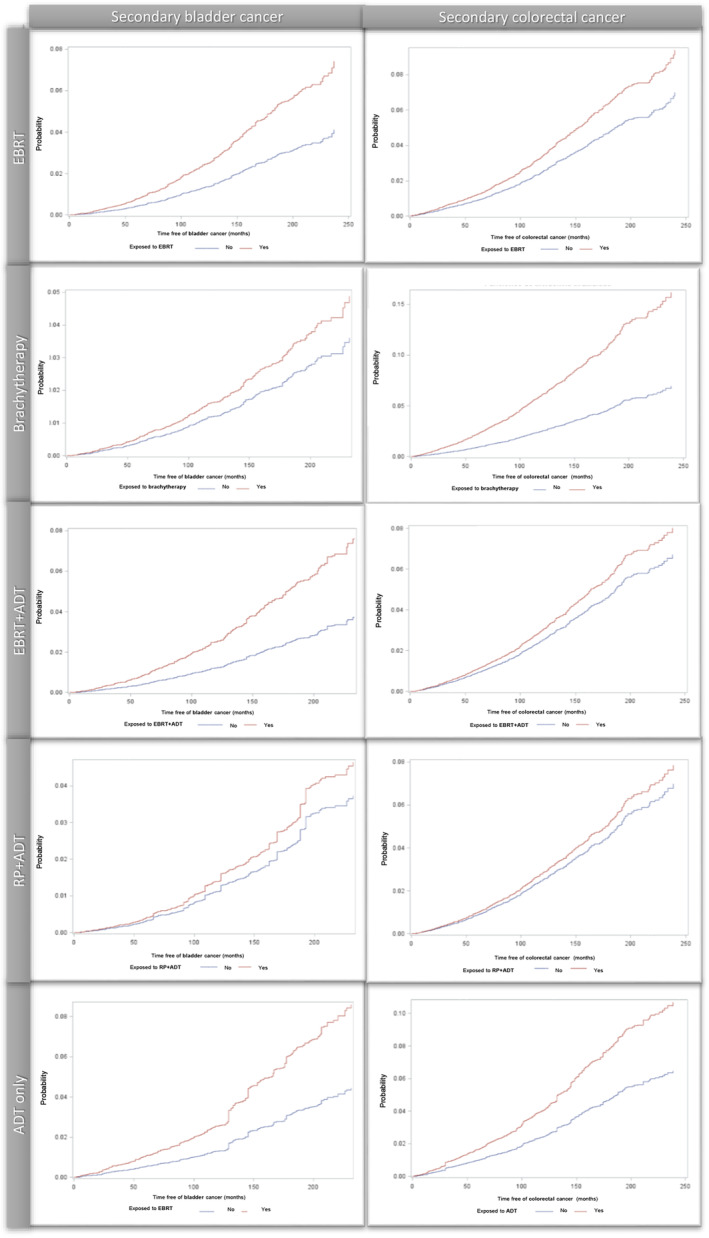
Cumulative incidence functions for secondary bladder cancer and colorectal cancer. ADT, androgen deprivation therapy; EBRT, external beam radiotherapy; RP, radical prostatectomy.

In addition, compared to RP, an increased risk of both secondary bladder and colorectal cancers was observed in patients treated with ADT alone (HR: 1.98, 95%CI: 1.69;2.31 and HR: 1.69, 95%CI: 1.49;1.92, respectively), yet no association was seen for RP + ADT (HR: 1.25, 95%CI: 0.68;2.30 and HR: 1.13, 95%CI: 0.77;1.68, respectively). Table 2 further provides the absolute 12‐month risks of experiencing bladder or colorectal cancer estimated from weighted analyses and corresponding CIF curves are presented in Figure 1.

## 4 DISCUSSION

We identified a significant association between the development of secondary bladder and colorectal cancers for EBRT alone or combined with ADT, compared to RP. These results align with the findings of previous Canadian studies showing an association between secondary cancers and RT.^18^, ^19^, ^25^ Bhojani et al.^18^ had shown the rates of bladder and rectal cancer were significantly higher in the EBRT group after 60 months of follow‐up (HR: 1.4; *p* = 0.02, and HR: 2.1; *p* < 0.001, respectively). Only the rectal cancer rates increased after 120 months (HR: 2.2; *p* = 0.003). More recently, Wallis et al.^19^ assessed the risk of RT complications in a cohort of 32,465 PCa patients, using a propensity score. While RT types were not differentiated, RT patients were more likely to be diagnosed with secondary malignancies than patients undergoing RP (HR: 2.44;95%CI: 1.16–5.14).

In our study, BT was significantly associated with colorectal cancer. The odds of a second cancer seem to vary depending on the RT type: EBRT is more often associated with increased odds compared to BT.^12^ Compared to RP, the incidence of rectum cancer seems to increase in men receiving EBRT alone and combined with BT (adjusted incidence rate ratio: 1.58 [95%CI:1.33;1.87] and 1.98 [95%CI: 1.50;2.61]) respectively.^26^ In a single institution retrospective study, Hamilton et al.^25^ compared the second malignancy incidence in PCa patients treated with BT relative to RP. Though the incidence increased, it was not statistically significant, which might be related to a shorter follow‐up time (up to 6 years) comparatively to our cohort.

Our study also found a significant association between ADT alone and both cancers. When combined to EBRT, the association remains present, even if marginally non‐significant when adjusted in colorectal cancer. Regarding secondary bladder cancer, our results differ from previous literature suggesting that ADT can reduce primary bladder cancer incidence, recurrence and specific mortality.^27^, ^28^ A retrospective Japanese study found a secondary bladder cancer occurred in 14 (2.2%), 5 (1.1%), and 0 (0%) PCa patients treated with RT, RP, and ADT, respectively. Though a low incidence was observed for RT and ADT, it should be considered that the follow‐up was short (50 months)^29^ and usually, the lag period (time elapsed for a tumor to be considered induced by radiation), involves a minimum of 5 years.^9^ Concerning colorectal cancer, Assayag et al^14^ conducted a populational study including 21,503 patients, of whom 184 were diagnosed with colorectal cancer during a mean follow‐up of 4 years. In this study, the use of ADT was not overall associated with an increased risk of colorectal cancer (HR:0.99, 95%CI:0.73;1.35). In contrast, a dose‐dependent effect of ADT associated with an increased risk of colorectal cancer was previously reported by Gillessen et al.^13^ Similarly, Lu and colleagues observed in 2015, an increased risk of colorectal cancer in patients diagnosed with PCa after 1980, when the use of ADT increased in Sweden. Among those who received treatment other than estrogen, the standardized incidence ratio was 1.37 (95%CI:1.29;1.45). No increased risk of colorectal cancer was found in patients receiving estrogen therapy.^30^ The authors concluded that ADT might be a causal factor for the observed increased risk. While evidence about the ADT effects is still scarce, our study provides a new insight supported by analyses with reduced risk of bias.

The assessment of secondary malignancies associated to RT for PCa encounters issues of diagnostic bias. As patients receiving RT are known to experience symptoms such as hematuria or increased bowel urgency, there is a potential for detection bias for bladder and colorectal cancers.^12^, ^29^ As observed here and elsewhere,^7^ older patients and/or with more comorbidities are more likely to receive RT than RP. Although using a propensity score cannot account for all comorbidities as a randomized controlled trial, it improves the estimation of the association of treatments by reducing the impact of treatment selection bias in observational data.^19^ In this sense, our data suggest an association between secondary cancers and the treatment received.

Among the strengths of this study, using provincial administrative databases allows the assessment of these treatments effects in actual clinical practice, without participants being under strict protocols. Also, patients were followed up for up to 16 years, providing a sufficient latency period to establish an association between the date of exposure to radiation and the development of a secondary cancer, commonly defined as a minimum of 5 years.^12^, ^18^ Lastly, the use of IPTW method allowed to minimize bias by balancing groups in terms of baseline variables. Nonetheless, this study has also limitations. Due to the use of administrative healthcare claims data, important information on confounders, comorbidities and other risk factors associated with cancers other than PCa was unavailable. Particularly, the lack of information on smoking status to evaluate the bladder cancer risk; and obesity, known to predispose patients to colon^31^ and PCas,^32^ which might bias the risk attributed to RT.^12^ Even if confounding variables were analyzed through appropriate statistical methods using propensity scores, it is possible that other unmeasured factors linking secondary malignancies to RT might have been missed.

## 5 CONCLUSION

This study found a significant association between secondary bladder cancer and EBRT, ADT, alone or incombination in PCa patients. The association of secondary colorectal cancer was also significant for patients receiving either EBRT, BT or ADT. The association of both cancers with RP remained unsignificant.

## AUTHOR CONTRIBUTIONS

**Patricia Grisel Quintana Bárcena:** Conceptualization (supporting); data curation (equal); formal analysis (equal); investigation (equal); methodology (equal); writing – original draft (lead); writing – review and editing (equal). **Armen Garo Aprikian:** Conceptualization (lead); funding acquisition (equal); project administration (equal); resources (equal); supervision (equal); validation (equal); writing – review and editing (equal). **Alice Dragomir:** Conceptualization (supporting); data curation (lead); formal analysis (equal); funding acquisition (equal); investigation (equal); methodology (equal); project administration (equal); resources (equal); supervision (lead); validation (equal); writing – review and editing (equal).

## FUNDING INFORMATION

Alice Dragomir is s supported by the Fonds de Recherche du Québec–Santé Research Scholar Junior 2 Grant. The study was partially supported by the Rossy Cancer Network Research Funds. Armen G. Aprikian is holder of the Richard Tomlinson Chair.

## CONFLICT OF INTEREST STATEMENT

None of the contributing authors have any conflicts of interest.

## ETHICS STATEMENT

Ethical approval for this study was obtained from the McGill University Health Center Ethics Board and the Comission d'accès à l'information du Québec before data was obtained from the RAMQ databases.

## Data Availability

The data that support the findings of this study are available on request from the corresponding author. The data are not publicly available due to privacy or ethical restrictions.
